# Central Dysmyelination in SSADH‐Deficient Humans and Mice

**DOI:** 10.1002/acn3.70148

**Published:** 2025-07-31

**Authors:** Itay Tokatly Latzer, Henry H. C. Lee, Edward Yang, Cesar Alves, Mariarita Bertoldi, Caitlyn Fung, Spencer V. Steele, Eren Kule, Zijie Jin, Alexander Rotenberg, Jean‐Baptiste Roullet, Phillip L. Pearl

**Affiliations:** ^1^ Department of Neurology Boston Children's Hospital, Harvard Medical School Boston Massachusetts USA; ^2^ School of Medicine, Faculty of Medical and Health Sciences Tel‐Aviv University Tel Aviv Israel; ^3^ Rosamund Stone Zander Translational Neuroscience Center Boston Children's Hospital Boston Massachusetts USA; ^4^ F.M. Kirby Neurobiology Center Boston Children's Hospital Boston Massachusetts USA; ^5^ Department of Radiology Boston Children's Hospital, Harvard Medical School Boston Massachusetts USA; ^6^ Department of Neuroscience, Biomedicine and Movement Sciences University of Verona Verona Italy; ^7^ Medical Microbiology & Immunology Department University of Wisconsin‐Madison Madison Wisconsin USA; ^8^ Department of Neuroscience DePauw University Greencastle Indiana USA; ^9^ School of Medicine Koç University Istanbul Turkey; ^10^ Department of Pharmacotherapy College of Pharmacy and Pharmaceutical Sciences, Washington State University Spokane Washington USA

**Keywords:** GABA, inherited metabolic disorders, myelin, succinic semialdehyde dehydrogenase, white matter

## Abstract

**Objectives:**

Succinic semialdehyde dehydrogenase deficiency (SSADHD) is an inherited metabolic disorder characterized by an accumulation of γ‐aminobutyric (GABA). In addition to its synaptic role as an inhibitory neurotransmitter, GABA also plays an important role in myelination. We aimed to investigate the relationship between GABA and myelination abnormalities in SSADHD patients and the mouse model.

**Methods:**

Brain MRIs performed on 44 individuals (23 with SSADHD and 21 healthy controls) were independently reviewed by two neuroradiologists and scored using a disease‐specific myelination scoring system. Inter‐rater reliability (IRR) was assessed by the intraclass correlation coefficient. Myelination scores of SSADHD individuals were correlated with clinical, biochemical, magnetic resonance spectroscopy, and genetic data. Additionally, we investigated the expression of myelin‐related genes in a mouse SSADHD model.

**Results:**

Dysmyelination in SSADHD patients was overall mild, but significantly greater than in healthy controls (*p* < 0.001). In SSADHD patients, lower myelination scores were significantly correlated with younger age (*R* = 0.775, *p* < 0.001) and higher plasma GABA (*R* = −0.722, *p* < 0.001) and γ‐hydroxybutyric acid (GHB) (*R* = −0.683, *p* = 0.001). In SSADH‐deficient mice, there was reduced expression of genes encoding myelin basic protein (*p* = 0.001), myelin‐associated oligodendrocyte basic protein (*p* = 0.001), and mitochondrial aspartate transporter (*p* = 0.025).

**Interpretation:**

Excessive GABA and GHB, which characterize SSADHD and are further pronounced in younger SSADHD individuals, may account for delayed oligodendrocyte maturation and altered myelination dynamics in this disorder. Studying the properties of dysmyelination in this unique disorder enhances our understanding of GABA's mediating role on myelination and may contribute to monitoring disease progression and managing other white‐matter neurological disorders.

**Trial Registration:**

NCT03758521

## 1 Introduction

Succinic semialdehyde dehydrogenase deficiency (SSADHD) (OMIM #271980) is an inherited metabolic disorder resulting from bi‐allelic *ALDH5A1* pathogenic variants [1, 2, 3, 4, 5, 6]. Its occurrence rate is rare, with an estimated worldwide prevalence ranging between 1/223,000 and 1/564,000 [7]. The primary metabolic defects in SSADHD involve impaired γ‐aminobutyric acid (GABA) catabolism, leading to the accumulation of GABA and other GABA‐related metabolites such as γ‐hydroxybutyrate (GHB) and γ‐guanidinobutyrate (GBA) [8, 9]. However, with increasing age and the constant synaptic GABA excess, GABA receptors are downregulated [10, 11]. The clinical presentation of SSADHD is wide, ranges in severity, and may have onset at different ages. Typically, though, symptoms appear in the first year of life [12, 13]. The predominant signs and symptoms of SSADHD include developmental delay, intellectual disability, hypotonia, movement disorders, epilepsy, and severe behavior problems and psychiatric traits [14, 15, 16]. Currently, existing treatments for SSADHD are symptomatic and supportive, and there are ongoing efforts to develop targeted enzyme and gene replacement therapies [17].

The common neuroimaging findings in SSADHD include magnetic resonance imaging (MRI) T2‐ and FLAIR‐weighted signal hyperintensities in the globus pallidi, subthalamic nuclei, and cerebellar dentate nuclei. Cortical and cerebellar atrophy [18] and subcortical white matter T2 and FLAIR hyperintensities have also been described [19, 20]. These findings are neither specific nor diagnostic of the disorder [21].

GABA has been shown to be crucial for myelination by controlling the development and function of oligodendrocyte progenitor cells (OPCs), which mature into myelin‐producing oligodendrocytes [22, 23, 24, 25, 26, 27]. Investigations in SSADH knock‐out (KO) (*Aldh5a1* ^−/−^) mice revealed they were characterized by elevated extracellular GABA levels, and accordingly, increased tonic GABAergic inhibition [28]. Impaired astrocyte and oligodendrocyte function were also demonstrated through a proteomic analysis in SSADH KO mice [29], as well as the downregulation of genes amenable to various myelination processes [30, 31]. Abnormal myelination was also demonstrated in post‐mortem analyses of an SSADHD patient [32, 33]. Taken together, the data suggest that myelination is impaired in SSADHD; however, a detailed description of the qualities of myelin and myelination in a larger set of SSADHD patients, and of the relationship between their myelination and other clinical and laboratory parameters, has yet to be reported.

SSADHD is not considered to be a classic hypomyelinating or demyelinating disease. However, considering the increased GABA levels characterizing this disorder, we hypothesized that the severity of myelin‐related alterations in SSADHD individuals would be associated with the degree of excess GABA. We investigated our hypothesis by assessing the correlation between MRI‐derived myelination scores and biochemical (plasma GABA and its metabolites), magnetic resonance spectroscopy (MRS), and genetic (expression of GABA‐related genes, bioinformatics and *in silico* analysis) metrics of SSADHD individuals. Additionally, we investigated the expression of myelin‐related genes in *Aldh5a1* ^*lox‐STOP*^ (inducible SSADH) mice. Our investigation aims to shed light on the interaction between GABA and its metabolites to myelination. Findings from this study may not only be used for prognostication and pending targeted therapeutics in SSADHD but also inform about myelin involvement in disorders of GABA catabolism or transport, risks related to medications targeting GABA mechanisms (e.g., vigabatrin), and treatment approaches to other encephalopathies with impaired myelination.

## 2 Methods

### 2.1 Data From Human SSADHD Individuals

This case–control study included genetically confirmed SSADHD participants and healthy, non‐related control subjects enrolled in the ongoing SSADHD Natural History Study [ClinicalTrials.gov ID: NCT03758521, approved by Boston Children's Hospital IRB (P00029917)], which commenced March 2019. Recruitment of SSADHD individuals for this study is done with the assistance of the SSADHD Association, and recruitment of healthy controls is done through the community. Enrollees arrive biennially for clinical visits, in which they undergo a series of tests, including medical and neurological examinations, neuroimaging, including magnetic resonance imaging (MRI) and magnetic resonance spectroscopy (MRS), in which cortical GABA levels are assessed. Biochemical and molecular testing is performed to assess GABA, GABA‐related metabolites (e.g., GHB), and GABA‐related gene expression. Genotype–phenotype correlations are performed using bioinformatics and *in silico* analyses of each participant's genotype and pathogenic variant combinations to determine their structural and functional impact on the SSADH enzyme.

#### 2.1.1 Assessment of Myelination

We adapted a myelination scoring system based on scoring systems designed and validated for adrenoleukodystrophy [34] and Pelizaeus‐Merzbacher disease [35]. Eighteen sampling areas reflecting typical myelination assessment regions [36, 37] were selected (Table 1). The main modification of the scoring system is an additional focus on those areas known to be abnormal in SSADHD, specifically periatrial white matter and basal ganglia, as well as inclusion of T2‐weighted imaging [18]. Of 18 areas studied (Table 1), each area was scored as “0” if completely lacking myelin, “1” if myelination is incomplete, and “2” if myelination is mature. The summation of individual scores yielded a total score, which was then expressed as a percentage of the age‐appropriate expected myelination, where 100% represented full myelination. The degree of dysmyelination was defined as follows: severe if the percentage was less than 50%, moderate if it ranged from 51% to 75%, and mild if it was between 76% and 100%. Additional measurements included the average thinnest periatrial white matter volume as well as corpus callosum anteroposterior length and thickness of the splenium and genu [38]. MRI scan review and myelination scoring were performed independently by two board‐certified pediatric neuroradiologists, both with additional specialization in inherited metabolic disorders (EY and CA), and blinded to the subjects' identities and clinical and genetic features. Non‐sedated MRIs were performed by a whole‐body 3‐Tesla MRI scanner (Siemens Skyra, Erlangen, Germany) with a 64‐channel phased array head coil. The MRI protocol included T1‐weighted MPRAGE sequences with 1 mm isotropic resolution, T2‐weighted fast spin echo sequences with 2 mm slice thickness and 0.4 mm in‐plane resolution, and fat‐suppressed 3D fluid‐attenuated inversion recovery sequences with 0.9 mm isotropic resolution. Where motion artifact was present, single‐shot T2 HASTE imaging was performed to enable measurement of critical parameters.

**TABLE 1:** Age‐based myelination scoring system adapted for SSADHD.

Months	0	1	2	3	4	5	6	7	9	11	12	15	18	24	36
Area sampled															
Middle cerebellar peduncle	1	1	2	2	2	2	2	2	2	2	2	2	2	2	2
Basal ganglia	2	2	2	2	2	2	2	2	2	2	2	2	2	2	2
Peri Rolandic cortex	1	2	2	2	2	2	2	2	2	2	2	2	2	2	2
PLIC	2	2	2	2	2	2	2	2	2	2	2	2	2	2	2
Corona radiata	1	2	2	2	2	2	2	2	2	2	2	2	2	2	2
Dorsal pons	2	2	2	2	2	2	2	2	2	2	2	2	2	2	2
Cerebellar WM		1	1	1	1	2	2	2	2	2	2	2	2	2	2
Dentate nucleus		1	2	2	2	2	2	2	2	2	2	2	2	2	2
Optic radiation			1	2	2	2	2	2	2	2	2	2	2	2	2
Hippocampus				1	1	1	1	1	1	1	1	1	1	1	1
Splenium CC						1	2	2	2	2	2	2	2	2	2
Genu CC						1	1	2	2	2	2	2	2	2	2
ALIC							1	1	2	2	2	2	2	2	2
Frontal central WM										1	1	2	2	2	2
Occipital peripheral WM											1	2	2	2	2
U‐fibers (frontal)												1	2	2	2
Frontal peripheral WM												1	2	2	2
Periatrial WM												1	1	1	1

*Note:* “0”‐ Myelin is completely lacking; “1”‐ Myelination is incomplete; “2”‐ Mature myelination.

Abbreviations: ALIC, Anterior limb of the internal capsule; CC, Corpus callosum; PLIC, Posterior limb of the internal capsule; WM, White matter.

#### 2.1.2 Plasma GABA and GHB

GABA was measured by high‐performance liquid chromatography‐electrospray ionization tandem mass spectrometry (HPLC‐ESI‐MS/MS) using GABA‐D_2_ as an internal standard [39]. GHB was measured using gas chromatography–mass spectrometry (GCMS) and ^2^H_6_‐GHB as an internal standard [40].

#### 2.1.3 Cortical GABA

Cortical GABA was measured as GABA/N‐acetyl aspartate (NAA) ratio using two MRS acquisitions (TR 1500 ms; TE 68 ms; bandwidth 1200 Hz) of single voxel GABA‐specific MEGA‐PRESS sequences (voxel size dimensions were 27 cm^3^; 30 × 30 × 30 mm). The initial acquisition included a 1.9 ppm‐arranged editing pulse, enabling selective refocusing of the 3.0 ppm GABA multiplet, and the sequential acquisition used a different location to spot the inversion, determining the J‐evolution of GABA. The basal ganglia were primarily sampled as the area mostly associated with T2‐weighted hyperintensities in SSADHD [18]. The posterior cingulate and occipital cortices were also systematically used for myelination scoring because these areas were proven to yield reliable MRS GABA measurements [41, 42, 43]. Spectroscopy data were processed by the LCModel9 software (version 6.3) [44].

#### 2.1.4 ALDH5A1 Gene Expression

To quantify *ALDH5A1* expression, we started by isolating RNA from whole blood using a PAXgene Blood miRNA Kit (QIAGEN, cat no. 763134, Hilden, Germany). We ensured the RNA was of sufficient quality and concentration using a Fragment Analyzer System Kit (cat. no. DNF‐472‐0500, Agilent, Santa Clara, CA) and the Qubit RNA HS assay kit (Invitrogen, cat. no. Q32855, Waltham, MA). For cDNA synthesis, we used the RT2 First Strand Kit within a custom 384‐well RT2 Profiler array (QIAGEN, Hilden, Germany). Quantitative polymerase chain reaction (qPCR) was then carried out on a CFX 384 (Bio‐Rad Laboratories, Hercules, CA). We normalized *ALDH5A1* expression to *GAPDH* and presented the results as 2^ΔCT^.

#### 2.1.5 Impact of ALDH5A1 Pathogenic Variants on SSADH Protein Structure and Function

The structural impact on the SSADH protein, derived from each participant's unique combination of variants, was assessed *in silico*, considering the amino acid substitutions, residue localization, microenvironment, and monomer interactions [45, 46]. The functional impact of the pathogenic variants on SSADH was assessed, taking into account the possibility of polypeptide chain truncation or amino acid substitutions, and using bioinformatics tools (Scale‐invariant feature transform (SIFT) and Polymorphism Phenotyping v2 (POLYPHEN2)). The stability of SSADH was estimated by Gibbs free energy change (ΔΔG, kcal/mol) assessed with CUPSAT [47]. Conservation analyses were carried out by the Consurf Web Server [48]. BindProfX [49] was utilized to understand whether there was impairment in the process by which monomers form dimers or tetramers according to differences in the type of ΔΔG values, calculated by an algorithm combining FoldX physics‐based potentials with conservation scores from pairs of protein–protein interaction surface sequence profiles.

### 2.2 Data From the *Aldh5a1* ^*lox‐STOP*^ Murine Model

#### 2.2.1 Brain Tissue Dissection and Total RNA Extraction

Hippocampus tissues were dissected from wild‐type (WT) (*n* = 9; 4M, 5F) and homozygous (HOM) (*n* = 15; 5M, 10F) mutant mice harboring the *Aldh5a1* ^*lox‐STOP*^ allele at postnatal days 18–19 (P18‐P19) [50]. The hippocampus was chosen as the sampling area for gene expression as it is more robust than other brain structures and is typically sampled for microdissection of murine brain tissue. Dissected tissues (~30 mg) were snap frozen by liquid nitrogen, followed by total RNA extraction using the QIAwave RNA mini kit (Qiagen). RNA quality and quantity were confirmed by Qubit RNA high sensitivity assay (Invitrogen). Reverse transcription of 2 μg of extracted total RNA was performed using a high‐capacity cDNA reverse transcription kit (Applied Biosystems).

#### 2.2.2 Quantitative Reverse Transcription‐Polymerase Chain Reaction (qRT‐PCR) Measures of Myelin‐Related Genes in Mouse

Quantitative PCR reactions were performed using the TaqMan Gene Expression Assay (ThermoFisher Scientific) with predesigned primers. The list of genes for which expression was estimated is provided in Table 2. *Actb* (encoding β‐actin) was used as a housekeeping gene. Gene expressions were calculated using the delta–delta CT method. qPCR reactions were performed in duplicate in a 96‐well assay plate. The CT values of each gene were averaged (CT_avg_), and delta CT values of each sample were calculated by subtracting the gene of interest's CT_avg_ from the housekeeping gene's CT_avg_. The geometric mean of control delta, reflected by the CT_avg_ from WT samples, was estimated, and delta–delta CT_avg_ values were calculated by subtracting delta CT_avg_ from control delta CT_avg_. Fold change of expressions was then calculated using the formula 2^−(delta–delta CT_avg_) for each sample, followed by conversion to % WT expressions.

**TABLE 2:** List of TaqMan gene expression assays in the mouse species.

Assay ID	Gene
Mm01266402_m1	*Mbp* (Myelin basic protein)
Mm02745649_m1	*Mobp* (Myelin‐associated oligodendrocytic basic protein)
Mm01339780_m1	*Mal* (Myelin and lymphocyte protein)
Mm00480867_m1	*Aspa* (Aspartoacylase)
Mm00489442_m1	*Slc25a13* (Solute carrier family 25, member 13^a^)
Mm02619580_g1	*Actb* (Actin, beta)

^a^ Also known as Citrin.

#### 2.2.3 Data Analysis

Data derived from investigations of SSADHD patients were analyzed using SPSS Statistics (IBM SPSS Statistics, Version 28, 2021, IBM Corp, Armonk, NY, USA). Categorical variables were reported as their relative frequencies. The normality of continuous variables was assessed via histograms and Kolmogorov–Smirnov testing. Data were reported as mean ±1 standard deviation (SD) (normal distribution) or medians and interquartile ranges (skewed distributions). Group comparisons and correlations were performed using either the Student *t*‐test and the Pearson correlation method or the non‐parametric Mann–Whitney test and Spearman's correlation analysis as appropriate. The intra‐class correlation coefficient (ICC) was used to estimate the inter‐rater reliability (IRR) of the myelination scores provided by the two MRI reviewers. ICC analyses were based on a “two‐way mixed” model and an “absolute agreement” type [51]. The ICC was interpreted as follows: ≤ 0.5 = poor; 0.5 < and ≤ 0.75 = moderate; 0.75 < and ≤ 0.9 = good; and > 0.9 = excellent. Statistical analysis for the myelin‐related gene expression in the SSADH‐deficient mice model was performed using a nonparametric Mann Whitney test and One‐Way ANOVA with multiple comparisons using the GraphPad Prism software (GraphPad, Boston, MA, USA). A *p*‐value ≤ 0.05 was considered significant for all analyses.

## 3 Results

The study included 44 individuals, 23 with SSADHD and 21 healthy controls. The IRR of the myelination score between the two MRI reviewers was “good” (ICC = 0.78, 95% CI [0.50–0.90]). A comparison of demographic, biochemical, and myelination‐related parameters of the study and control groups showed that the SSADHD group had significantly decreased SSADHD myelination scores (*p* < 0.001), as well as periatrial white matter volume (*p* < 0.001), and corpus callosum genu thickness (*p* < 0.001) and anterior–posterior length (*p* = 0.05). Plasma GABA and GHB, as well as cortical GABA, were significantly higher in SSADHD individuals compared to controls (*p* < 0.001 for all three) (Table 3). The groups did not differ in age and sex distribution.

**TABLE 3:** Comparison of demographic and MRI‐derived myelination‐related features of SSADHD individuals and healthy controls.

Characteristic	SSADHD individuals	Healthy controls	*p*
	*N* = 23	*N* = 21	
Age, mean ± SD	12.7 ± 9.6	18.2 ± 9.8	0.06
Sex (male/female)	12/11	10/11	0.50
Myelination score	92.1 ± 2.6	99.7 ± 0.6	**< 0.001**
Periarterial white matter volume	6.4 ± 1.1	8.3 ± 1.3	**< 0.001**
CC Genu thickness, mm	10.0 ± 1.2	11.8 ± 1.9	**< 0.001**
CC Splenium thickness	10.0 ± 1.6	10.9 ± 1.4	0.07
CC anterior–posterior length, mm	68.1 ± 6.3	71.5 ± 4.7	**0.05**
Plasma GABA, μM/L	3.0 (2.1–3.6)	0.9 (0.9–1.0)	**< 0.001**
Plasma GHB, μM/L	217 (81–377)	0.5 (0–1.2)	**< 0.001**
MRS GABA/NAA	0.20 (0.17–0.23)	0.08 (0.07–0.09)	**< 0.001**

*Note:* Bold indicates significant statistical power.

Abbreviations: CC, Corpus callosum; GABA, γ‐aminobutyric acid; GHB, γ‐hydroxybutyrate; MRS, Magnetic resonance spectroscopy; SD, Standard deviation; SSADHD, Succinic semialdehyde dehydrogenase deficiency.

**FIGURE 1:**
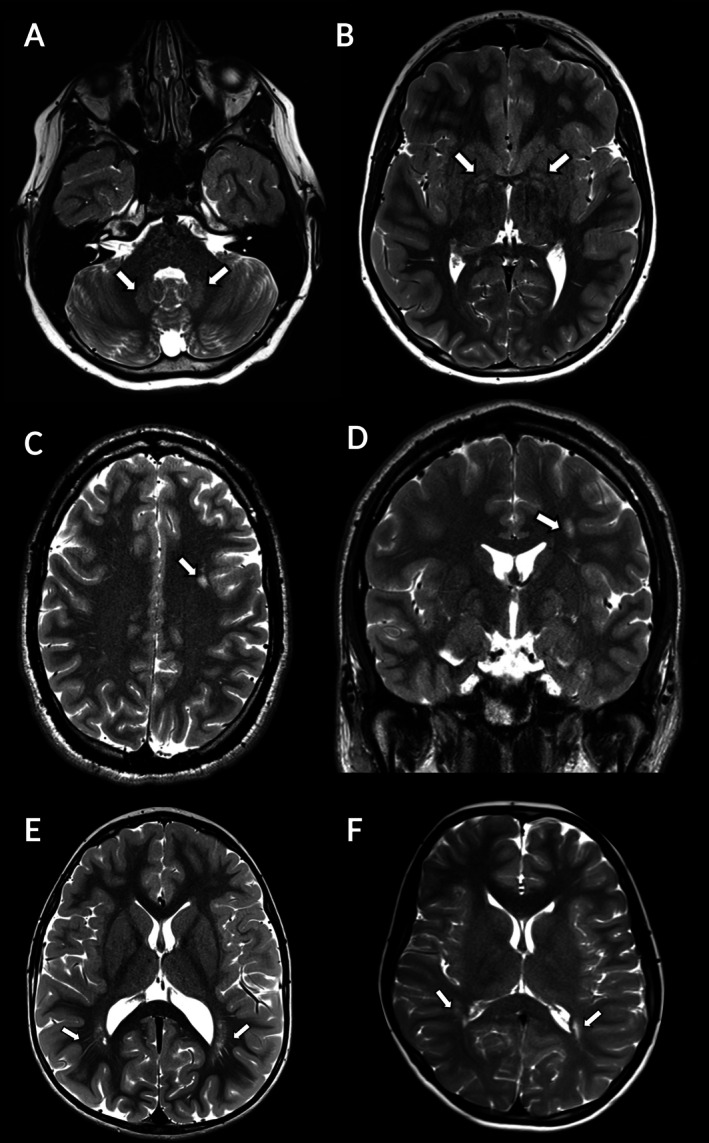
Myelination abnormalities in individuals with succinic semialdehyde dehydrogenase deficiency (SSADHD), as demonstrated by signal hyperintensities (white arrows) in T2‐weighted MRI images in the dentate nuclei (A) and globus pallidi (B) of a 9‐year old individual, subcortical white matter of the left frontal lobe (C, D) of a 24 year‐old individual, and posterior peri‐atrial regions (E, F) of a 3‐year old individual.

Notably, within the SSADHD group only, males and females did not differ in their myelination scores (*p* = 0.24), nor the periatrial white matter volume (*p* = 0.67), corpus callosum genu thickness (*p* = 0.64), splenium thickness (*p* = 0.43), and anterior–posterior length (*p* = 0.37). Each participant from the SSADHD group had at least one area of abnormal myelination, with none of the participants' scores denoting severe (below 50%) or moderate (below 75%) dysmyelination. The lowest myelination score was 87.5% (Table 4). The areas where dysmyelination was most commonly identified were the dentate nucleus, basal ganglia, periatrial white matter, corona radiata, and frontal peripheral white matter (Figure 1). Within the SSADHD group, lower myelination scores significantly correlated with younger age (*R* = 0.775, *p* < 0.001). As shown in Table 4, participants #1–8, whose myelination scores were in the lowest quartile, did not differ from the rest of the participants in the upper three quartiles in genetic and clinical characteristics. For example, patients #1 and #2, who had the lowest myelination scores, did not have seizures, and the four patients with the highest myelination scores (#20, #21, #22, and #23) all had epilepsy. However, the myelination scores were significantly correlated with higher plasma GABA (*R* = −0.722, *p* < 0.001) and plasma GHB (*R* = −0.683, *p* = 0.001) and had a correlative tendency to higher MRS GABA/NAA (*R* = −0.399, *p* = 0.07). Notably, within the group of SSADHD subjects, lacking or having a truncated SSADH protein did not relate to dysmyelination, nor did the structural or functional impairment in SSADH derive from the specific gene variant combination. Additionally, we did not observe a specific relationship between the myelination scores and the medications with which SSADHD individuals were treated.

**TABLE 4:** Myelination scores and their relation to clinical, genetic, biochemical, and MRS features.

#	Myelination score	Dysmyelinated areas	Age (years)	Pathogenic variant 1	Pathogenic variant 2	Structure/function affected in protein (SSADH)	ALDH5A1 expression (2^ΔCT^)	Epilepsy	Movement disorders	Behavior problems	Medications	Plasma GABA (μM/L)	Plasma GHB (μM/L)	MRS GABA/NAA
1	87.5	DN, BG, FUF, FPWM	1.9	c.1226G>A	c.1323dupG	Stability/folding	0.0146	No	No	No	—	4.46	1430.8	0.25
2	88.9	DN, BG PAWM, CR	10.4	c.612G>A	c.1234C>T	No protein	0.0117	No	Yes	Yes	—	3.22	—	0.21
3	90.3	DN, BG PAWM	8.4	c.612G>A	c.1234C>T	No protein	0.0082	No	Yes	Yes	—	3.5	—	0.21
4	90.3	DN, BG PAWM	3.6	c.1015‐2A>C	c.1597G>A	Oligomerization	0.0155	No	No	No	β‐agonist	3.64	340.9	0.235
5	90.3	DN, BG PAWM	9.7	c.967_968dupCA	c.1597G>A	Oligomerization	0.0053	Yes	Yes	Yes	AAP SSRI	3.72	139.7	0.235
6	90.3	DN, BG PAWM	1.8	c.526G>A	c.1226G>A	Folding/stability and oligomerization	0.0526	No	No	No	PPI	4.55	1335.4	0.195
7	90.3	DN, BG PAWM	1.2	c.612G>A	c.1597G>A	Oligomerization		No	No	No	—	4.61	4323.2	0.225
8	90.3	DN, BG, CR	6.4	c.612G>A	c.870+3delA	No protein	0.0187	Yes	No	Yes	α‐agonist	3.85	217.5	0.17
9	91.7	DN, BG PAWM	24.3	c.612G>A	c.612G>A	No protein	0.0091	No	Yes	Yes	AAP SSRI	2.06	81.2	0.155
10	91.7	DN, BG PAWM	10.02	c.608C>T	c.608C>T	Catalysis and NAD+ binding	0.0253	Yes	Yes	Yes	ASM Stimulant	3.13	302	0.175
11	91.7	DN, BG	5.34	c.612G>A	c.803G>A	Catalysis	0.0095	No	No	Yes	—	2.94	228	0.28
12	91.7	DN, BG PAWM	4.33	c.278G>T	c.1015‐3C>G	Stability/folding	—	Yes	Yes	Yes	AAP	4.8	1041.2	0.24
13	91.7	DN, BG	16.31	c.754G>T	c.754G>T	Stability/folding	0.0386	No	No	Yes	—	2.06	99.1	0.175
14	91.7	DN, BG	13.38	c.754G>T	c.754G>T	Stability/folding	0.0272	Yes	No	Yes	ASM	2.56	119.8	0.16
15	91.7	DN, BG	12.82	c.1388del	c. 803G>A	Catalysis	—	No	Yes	Yes	—	—	—	0.2
16	91.7	DN, BG	13.91	c.1005C>A	c.1015‐2A>C	Catalysis and slightly in folding	—	Yes	No	Yes	ASM	—	—	0.27
17	93.1	DN, BG	9.63	c.612G>A	c.1321G>A	Stability/folding	0.0164	No	No	Yes	—	2.53	270.6	0.19
18	93.1	DN, BG	8.79	c.1226G>A	c.1226G>A	Stability/folding	0.0354	No	No	Yes	β‐agonist CS inhaler	3.55	377.9	—
19	93.1	DN, BG	24.90	c.754G>T	c.754G>T	Stability/folding	0.0370	Yes	Yes	No	—	2.18	74.5	0.19
20	95.8	DN, BG	23.28	c.612G>A	c.1402+2T>C	No protein	0.0161	Yes	No	Yes	ASM Stimulant	1.97	52.7	0.205
21	95.8	DN	27.06	c.612G>A	c.803G>A	Catalysis	0.0135	Yes	Yes	Yes	ASM Stimulant	1.66	52.7	0.23
22	97.2	DN, PAWM	16.21	c.104_127 del	c.1015‐2A>C	No protein	0.0096	Yes	Yes	Yes	ASM Stimulant	1.19	106.5	0.15
23	98.6	DN	39.68	c.612G>A	c.1015‐2A>C	No protein	0.0059	Yes	Yes	Yes	ASM SSRI	1.44	60.8	0.19

Abbreviations: AAP, Atypical antipsychotic; ALDH5A1, Aldehyde dehydrogenase 5 family member A1; ASM, Antiseizure medication; BG, Basal ganglia; CR, Corona radiata; CS, Corticosteroid; CT, Cycle threshold; DN, Dentate nucleus; FPWM, Frontal peripheral white matter; FUF, Frontal U‐fibers; GABA, γ‐aminobutyric acid; GHB, γ‐hydroxybutyrate; MRS, Magnetic resonance spectroscopy; NAA, N‐acetyl aspartate; NAD, Nicotinamide adenine dinucleotide; PAWM, Periatrial white matter; PPI, Proton pump inhibitor; SSADH, Succinic semialdehyde dehydrogenase; SSRI, Selective serotonin reuptake inhibitor.

### 3.1 Myelin‐Related Genes in the Aldh5a1^lox‐STOP^ Murine Model

**FIGURE 2:**
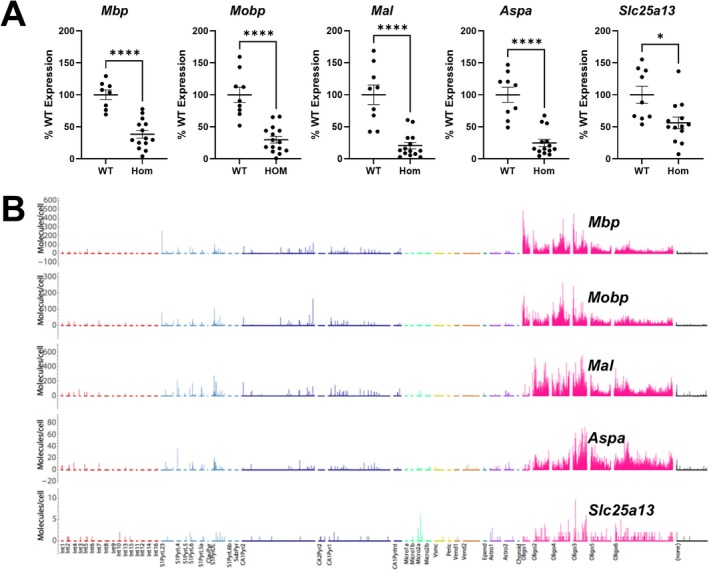
Expressions of myelin‐related genes in the hippocampus of *Aldh5a1* ^*lox‐STOP*^ mice. (A) qRT‐PCR results of gene expressions comparing wild type (WT) and homozygous (HOM) mice at postnatal day P18‐19. Gene names are listed on the top. Individual data points are shown. Data are presented as means and error bars are standard error of the mean (SEM). ****p < 0.0001, *p < 0.05, Mann–Whitney test. (B) Single‐cell RNA‐seq database search results of myelin‐related genes. Data is represented as molecules per cell, grouped according to cell type. Note these genes have a high expression in oligodendrocytes ‘Oligo’ groups in pink. (Credit: Linnarsson lab, https://linnarssonlab.org/cortex/).

**FIGURE 3:**
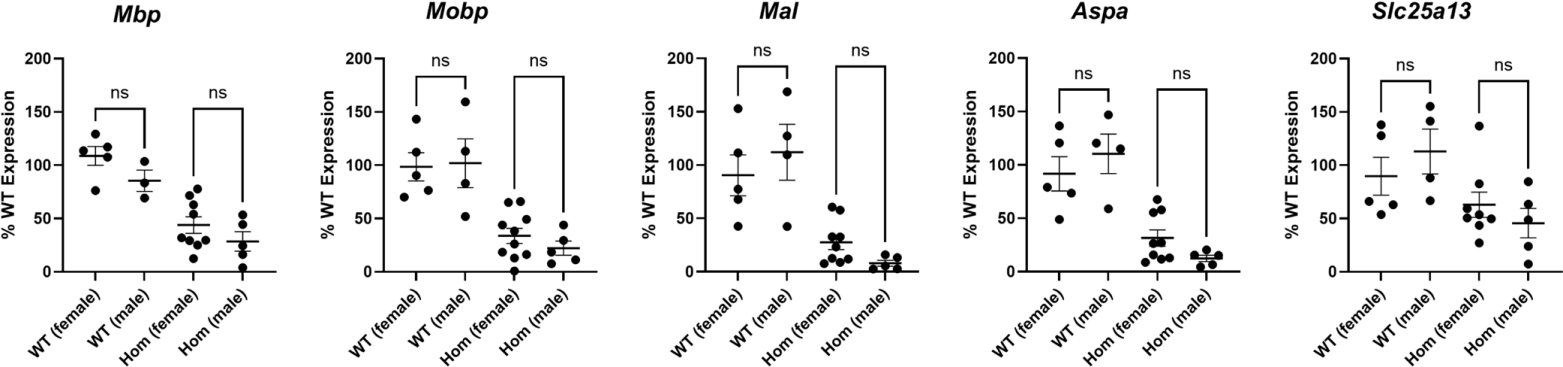
Comparisons of myelin‐related gene expressions between female and male *Aldh5a1* ^*lox‐STOP*^ mice (further detailed in Table S1). ns, not significant.

Data on 24 mice were included: 9 WT (four males and five females at P18‐P19) and 15 homozygous (HOM) (five males and ten females at P18‐P19). Transcript expressions of the following genes were significantly reduced in the hippocampus of HOM *Aldh5a1* ^*lox‐STOP*^ mice when compared to the WT mice (data are represented as % WT control): (1) *Mbp*‐ WT: 100% ± 7.55%, HOM: 38.37% ± 6.06%, *p* < 0.0001; (2) *Mobp*‐ WT: 100% ± 11.71%, HOM: 29.83% ± 5.26%, *p <* 0.0001; (3) *Mal*‐ WT: 100% ± 15.21%, HOM: 20.54% ± 5.06%, *p* < 0.0001; (4) *Aspa*‐ WT: 100% ± 11.81%, HOM: 24.71% ± 5.49%, *p* < 0.0001; (5) *Slc25a13*‐ WT: 100% ± 13.37%, HOM: 56.27% ± 8.99%, *p* = 0.0105. (Figure 2A). A single‐cell RNA‐seq database search revealed that all the aforementioned genes are expressed in oligodendrocytes (Figure 2B). Notably, no significant differences in gene expression were observed between female and male mice (Figure 3, Table S1).

## 4 Discussion

We report significantly more dysmyelination in SSADHD individuals compared to healthy controls, with more impairment in younger SSADHD patients than in older ones, who are furthermore characterized by higher plasma and cortical GABA levels. Our investigation was based on a semiquantitative myelination scoring system adapted from other leukodystrophies, employed by two neuroradiologists who were blinded to the participants' clinical characteristics and demonstrated good interrater reliability. While SSADHD is not primarily a leukoencephalopathy, evidence derived from several sources points to impaired myelination in this disorder. Prolonged latencies using somatosensory evoked fields support impaired white matter axonal conduction [52]. Proof‐of‐concept MRI G‐ratio mapping, a more specific method for determining myelin integrity, showed that myelin was diffusely depressed in an SSADHD individual compared to a healthy control [53], and a post‐mortem analysis on an SSADHD patient identified mild to moderate white‐matter astrogliosis in several cortical regions [32, 33].

Additionally, we evaluated the expression of myelin‐related genes in the *ALDH5A1* ^lox‐STOP^ mouse model of SSADHD. Our results showed that myelin and oligodendrocyte‐associated gene expressions are significantly reduced in SSADH‐deficient mice. These data are consistent with previous findings using a global SSADH knockout mouse model, describing that compared to age‐matched wild‐type littermates, SSADH knockout mice exhibit significant downregulation of myelin‐related genes (*Mbp*‐ Myelin basic protein, *Mobp*‐ Myelin associated oligodendrocytic basic protein, and *Mal*‐ Myelin and lymphocyte protein, T‐cell differentiation protein), especially in the hippocampus [30]. MBP is a microtubule stabilizing protein in differentiated oligodendrocytes, essential for myelin integrity [54]. MOBP plays a key role in the maturation steps of myelin formation and sheath compaction [55]. MAL is a key regulator of myelin proteolipid protein PLP [56]. Reduced expressions of these genes suggest an impaired myelination process in SSADH‐deficient mice. In addition, we identified two genes associated with myelinating oligodendrocytes that are significantly reduced and have not been reported in the global SSADH knockout mouse model (*Aspa*‐ Aspartoacylase, and *Slc25a13*‐ Mitochondrial aspartate–glutamate carrier 2). Aspa immunoreactivity in rodent white matter implies a role in myelination [57]. Further, Aspa deficiency affects the development of oligodendrocytes and leads to myelination impairment in mice, as demonstrated in a Canavan disease model [58, 59]. The mitochondrial aspartate–glutamate carrier 2 (AGC2, also termed Citrin, encoded by *Slc25a13*) is expressed in oligodendrocytes. Interestingly, reduced hippocampus *Slc25a13* has been associated with anxiety or depression‐like behaviors in mice, highlighting the role of mitochondrial dysfunction in pathologic behaviors [60]. In addition, we found that these gene expressions are similarly reduced in males and females mutant mice, consistent with the human data showing myelination‐related neuroimaging findings were indifferent between sexes. These data, indicating alterations in oligodendrocyte physiology and myelination in SSADH‐deficient mice, provide additional insights within the context of dysmyelination in SSADHD. Notably, several factors could explain why myelination abnormalities were not specifically observed in the hippocampi of individuals with SSADHD. These include variations in remaining enzyme activity between humans with SSADHD and SSADH‐deficient mice, as well as differences in the methodologies used to assess myelination in their studies. However, a post‐mortem analysis on an SSADHD patient did reveal white matter predominant mild to moderate astrogliosis in the hippocampi [32, 33].

From a molecular perspective, impairment of myelination in SSADHD may be accounted for by pathologically elevated concentrations of GABA. Over the last 40 years, a growing body of evidence has demonstrated that GABA plays a crucial mediating role in the synthesis and maintenance of central and peripheral myelin. Initially, GABA‐evoked responses were shown to occur in spinal cord oligodendrocytes of murine models [61], and this was confirmed by follow‐up studies [62, 63]. These and other reports suggested that GABAergic activity is essential for myelination, axon recognition, and migration of OPCs during their early stages of maturation [64, 65, 66]. Later, it was demonstrated in vitro and in vivo that the GABA effect on OPCs is lessened or changed as they become oligodendrocytes [25, 67, 68, 69], and that GABA elicits a dissimilar reaction in the different subgroups of oligodendrocytes [27]. GABA has also been shown to participate in the regulation of in remyelination processes [70, 71], including affecting the Src‐family kinases, which perform different roles throughout the origins and development of oligodendrocytes [22]. The evidence describing the relationship between oligodendrocytes and GABA indicates that GABAergic activity promotes myelination through diverse and intricate mechanisms. Hence, myelination may be altered by the inherent metabolic features of SSADHD, consisting of an excess of GABA, which diminishes relatively during early adolescence but remains high compared to healthy individuals. Notably, neither plasma nor cortical GABA was associated with the type of SSADH protein dysfunction [12], and therefore, it is unsurprising that an association between any structural or functional SSADH protein impairment and dysmyelination was not found in the current study.

Several investigations partly support the above notion. GABA administered endogenously to murine models was shown to increase the proliferation rate of OPCs and, by that, disrupt their maturation and myelin synthesis, an effect that was reversed by the provision of a GABA_A_ receptor antagonist [26]. An increment of GABA has been proven to have a profound neuroprotective role after cerebral ischemic stroke, as it inhibits neuronal circuits whose excitation may lead to adverse impacts on the regenerating cortex, and by promotion of brain‐derived neurotrophic factor (BDNF) expression and other beneficial immunomodulatory effects [72, 73]. However, the excessive GABA seen post‐stroke may also account for delayed oligodendrocyte maturation, altering the myelin dynamics required for proper myelination [74, 75]. Lastly, abnormal GABAergic activity (specifically, in the sensorimotor cortex) has been implicated in the progression of the demyelinating disease multiple sclerosis (MS) [76, 77, 78].

Our results indicate that significantly more myelination abnormalities were observed in younger individuals with SSADHD than in older ones. Myelin maturation may be delayed in SSADHD due to the higher GABA concentrations characterizing the early course of this disorder [50]. As these individuals age, the physiologic mechanisms by which myelin is produced and maintained may be recovered. Overall, these findings suggest a delayed developmental program in myelin and possibly oligodendrocytes in patients with SSADHD. These assumptions are based on separate data gathered from a relatively large number of SSADHD individuals at different age points. Revaluation and validation of these assumptions will be one of the aims of the continuing and decade‐long SSADHD Natural History Study, which, in addition to its current focus, will incorporate assessments such as MRI G‐ratio mapping and gene expression assessment of GABA_A_ receptors. The disease‐specific semiquantitative myelination scoring system used in this current study can be useful for subgrouping individuals in clinical trials, prognostication, and monitoring during targeted treatment trials in the future.

## 5 Conclusions

In the largest study of myelination abnormalities in individuals with SSADHD to date, we report that radiographic assessment of myelin showed significantly more dysmyelination compared to matched healthy controls. These findings indicate that myelin alterations in SSADHD are typically mild and primarily affect younger individuals in this population, who also have higher GABA levels than their older counterparts. These findings were achieved by a modified leukodystrophy myelination scoring system conducted by two neuroradiologists with good inter‐rater reliability. Our outcomes were complemented by demonstrating a decreased amount of myelin‐related gene expression in the SSADHD murine model (*Aldh5a1* ^lox‐STOP^ mice) compared to wild‐type littermates. Based on the outcomes of the current investigation, we anticipate near‐future studies that incorporate longitudinal data and MRI G‐ratio measurements in a larger sample of SSADHD individuals. Measuring the myelin dynamics in a disorder characterized by unique metabolic properties, including supraphysiological GABA concentrations, advances our understanding of the regulatory role GABA exerts on myelination. This may be beneficial for monitoring disease progression and future clinical trials of SSADHD and may also inform about myelin involvement in other disorders of GABA catabolism or GABA transport, risks to myelin integrity associated with GABA‐related medications, and managing other demyelinating clinical conditions.

## Author Contributions

I.T.L., H.H.C.L., A.R., and P.L.P. conceived and designed the study. I.T.L., E.Y., C.A., M.B., A.R., J.‐B.R., and P.L.P. were responsible for collecting and analyzing human data. H.H.C.L., C.F., S.V.S., E.K., and Z.J. collected and analyzed data from the murine models. I.T.L., H.H.C.L., and P.L.P. wrote the initial draft of the manuscript. All authors contributed to editing the manuscript by providing critical feedback and approved the final version.

## Conflicts of Interest

The authors I.T.L., E.Y., C.A., M.B., C.F., S.V.S., E.K., J.B.R., and P.L.P. have no relevant financial or non‐financial interests to disclose. The authors A.R. and H.H.C.L. are co‐founders and have equity in Galibra Neuroscience Inc., which develops treatments for SSADH deficiency, including gene replacement therapy mentioned in this study.

## Supporting information


**Table S1:** Sex‐based comparison of myelin‐related gene expression in wild‐type and homozygous mutant *Aldh5a1* ^lox‐STOP^ mice.

## Data Availability

The data that support the findings of this study are available on request from the corresponding author. The data are not publicly available due to privacy or ethical restrictions.
