# Supplementary material for: Central Dysmyelination in SSADH‐Deficient Humans and Mice
Source: Ann Clin Transl Neurol. 2025 Jul 31;12(11):2193–205. doi: 10.1002/acn3.70148 (PMC12623846; doi:10.1002/acn3.70148)
Supplement: Supplementary file 1 — Table S1: Sex‐based comparison of myelin‐related gene expression in wild‐type and homozygous mutant Aldh5a1 lox‐STOP mice. [file ACN3-12-2193-s001.docx]

**Supplemental Table 1. Sex-based comparison of myelin-related gene expression in wild-type and homozygous mutant *Aldh5a1*^lox-STOP^ mice.**

|  | **ANOVA** | | **Gene expression levels, Mean±SEM (%WT)** | | | |
| --- | --- | --- | --- | --- | --- | --- |
| **Gene** | **F** | **P value** | **WT**  **(females)** | **WT**  **(males)** | **HOM**  **(females)** | **HOM**  **(males)** |
| *Mbp* | 15.65 | <0.0001 | 108.7±8.87% | 85.42±9.96% | 43.85±7.72% | 28.5±9.06% |
| *Mobp* | 12.35 | <0.0001 | 98.5±13.32% | 101.9±22.9% | 33.69±7.07% | 22.12±6.59% |
| *Mal* | 12.25 | <0.0001 | 90.38±19.13% | 112±26.29% | 27.52±6.76% | 7.99±2.76% |
| *Aspa* | 15.39 | <0.0001 | 91.72±16.07% | 110.4±18.49% | 31.51±7.58% | 12.46±2.99% |
| *Slc25a13* | 3.154 | 0.0503 | 89.69±17.79% | 112.9±21.11% | 62.93±11.88% | 45.6±13.77% |

ANOVA- Analysis of variance; HOM- Homozygous; SEM- Standard error mean; WT- Wild type.
